# Immunosuppression and COVID-19 infection in British Columbia: Protocol for a linkage study of population-based administrative and self-reported survey data

**DOI:** 10.1371/journal.pone.0259601

**Published:** 2021-11-19

**Authors:** Ana Michelle Avina-Galindo, Zahra A. Fazal, Shelby Marozoff, Jessie Kwan, Na Lu, Alison M. Hoens, Jacek Kopec, Diane Lacaille, Hui Xie, Jonathan M. Loree, J. Antonio Avina-Zubieta, on behalf of the SCOUT team

**Affiliations:** 1 Arthritis Research Canada, Vancouver, Canada; 2 Faculty of Land and Food Systems, University of British Columbia, Vancouver, British Columbia, Canada; 3 Faculty of Science, University of British Columbia, Vancouver, British Columbia, Canada; 4 Department of Physical Therapy, University of British Columbia, Vancouver, British Columbia, Canada; 5 School of Population and Public Health, University of British Columbia, Vancouver, British Columbia, Canada; 6 Division of Rheumatology, Department of Medicine, University of British Columbia, Vancouver, British Columbia, Canada; 7 Faculty of Health Sciences, Simon Fraser University, Burnaby, British Columbia, Canada; 8 Division of Medical Oncology, Department of Medicine, University of British Columbia, Vancouver, British Columbia, Canada; 9 BC Cancer, Vancouver, British Columbia, Canada; PLOS ONE, UNITED STATES

## Abstract

**Introduction:**

Cases of the novel coronavirus disease (COVID-19) continue to spread around the world even one year after the declaration of a global pandemic. Those with weakened immune systems, due to immunosuppressive medications or disease, may be at higher risk of COVID-19. This includes individuals with autoimmune diseases, cancer, transplants, and dialysis patients. Assessing the risk and outcomes of COVID-19 in this population has been challenging. While administrative databases provide data with minimal selection and recall bias, clinical and behavioral data is lacking. To address this, we are collecting self-reported survey data from a randomly selected subsample with and without COVID-19, which will be linked to administrative health data, to better quantify the risk of COVID-19 infection associated with immunosuppression.

**Methods and analysis:**

Using administrative and laboratory data from British Columbia (BC), Canada, we established a population-based case-control study of all individuals who tested positive for SARS-CoV-2. Each case was matched to 40 randomly selected individuals from two control groups: individuals who tested negative for SARS-CoV-2 (i.e., negative controls) and untested individuals from the general population (i.e., untested controls). We will contact 1000 individuals from each group to complete a survey co-designed with patient partners. A conditional logistic regression model will adjust for potential confounders and effect modifiers. We will examine the odds of COVID-19 infection according to immunosuppressive medication or disease type. To adjust for relevant confounders and effect modifiers not available in administrative data, the survey will include questions on behavioural variables that influence probability of being tested, acquiring COVID-19, and experiencing severe outcomes.

**Ethics and dissemination:**

This study has received approval from the University of British Columbia Clinical Research Ethics Board [H20-01914]. Findings will be disseminated through scientific conferences, open access peer-reviewed journals, COVID-19 research repositories and dissemination channels used by our patient partners.

## Introduction

The first case of the novel coronavirus disease (COVID-19) in Canada was recorded on January 25th 2020, followed by the first case in the province of British Columbia (BC) on January 28th 2020 [1]. Since then, the World Health organization has declared the COVID-19 outbreak as a global pandemic (March 11th 2020) followed by the province declaring a state of emergency in the same month [1]. In May 2021, BC saw a decrease in the incidence rate from 137 cases per 100,000 to 30 cases per 100,000, an 80% decrease in incidence rate from the peak in the previous month [2]. However, evidence-informed policies and clinical care guidelines are necessary to ensure that patients from vulnerable groups continue to receive appropriate care including preventive measures such as priority vaccination [3, 4]. One such priority population is individuals with compromised immune systems such as those with autoimmune diseases, cancer, organ transplants, disease-induced immunosuppression (e.g., chronic kidney disease requiring dialysis/hemodialysis) and those receiving immunomodulatory medications.

Since the start of the pandemic, more than 330 trials have been recorded to target the specific inflammatory pathways that result in the severe manifestations of COVID-19 (e.g., cytokine storm) [5]. The potential treatments in these trials had included immunomodulators (e.g., minocycline) and immunosuppressive therapies (e.g. chemotherapy or monoclonal antibodies against TNF, IL-1, and IL-6), which are regularly used to treat patients with cancer, autoimmune diseases and organ transplants [5]. However, the literature on immunosuppressant and immunomodulatory agents (IIAs) offers mixed evidence, with some drugs increasing the risk of experiencing serious outcomes of COVID-19 while others show a protective effect [6]. A scoping review by Fung & Babik found that some immunocompromised patients have a higher risk of experiencing severe outcomes of COVID-19 such as those with cancer or organ transplants, while others taking biologics have a lower risk of severe outcomes [7]. Similarly, Gianfrancesco et al. found that within patients with rheumatic diseases, IIA medications such as corticosteroids are associated with an increased risk of hospitalization for COVID-19, while tumour necrosis factor inhibitors (anti-TNF) use is associated with lower odds of hospitalization [6]. However, a more recent study has shown that the risk of experiencing severe outcomes of COVID-19 in immunosuppressed patients is not statistically significant once factors such as race, smoking and comorbidities are adjusted for [8].

In addition to the contradictory findings regarding IIA use, there is a dearth of knowledge surrounding whether demographic variables, lifestyle choices and COVID-19 preventative behaviours impact the risk of COVID-19 infection among immunocompromised individuals either as confounders or as effect modifiers. For example, individuals with immunosuppression may perceive themselves to be at a higher risk for infection or severe outcomes of COVID-19 and thus be more vigilant and adherent to preventative measures (e.g., physical distancing, minimizing exposure to public transportation, wearing face masks). The significance of sociodemographic variables was shown by Li et al. and Kumar et al. who noted the difference in risk of infection based on factors such as age, sex, ethnicity, marital and employment status [9, 10]. Li et al. provided evidence that these factors were associated with the frequency of preventative behaviors, testing, and thus the risk of COVID-19 infection [9]. Unfortunately this type of information is difficult to acquire. As a result, despite the rich source of health data available in administrative health databases, there is insufficient information to fully characterize the relationship between immunosuppression and COVID-19 without accounting for clinical and behavioural variables.

To address this, we will invite a subsample of our study population assembled using administrative health data, to complete a survey that will be linked to their administrative data. The survey will gather covariates not available in administrative data including the likelihood of being exposed to or tested for SARS-CoV-2 to address potential confounding bias in the relationship between immunosuppression and COVID-19 risk. We will use a case-control design to assess the relative risk of COVID-19 infection in patients with and without immunosuppression after adjusting for relevant confounders. Furthermore, we will use a population-based cohort design to assess the risk of hospitalization, intensive care unit (ICU) admissions, and death in patients with and without immunosuppression who got COVID-19.

## Methods

### Data source

Publicly funded healthcare is available to all residents of the province of British Columbia (BC), Canada (population ~5.1 million) [11]. Population Data BC uses population-based linkable administrative data files to capture all provincially funded healthcare services, including all outpatient medical visits (to specialists and primary care physicians), interventions, investigations [12], cancer diagnoses [13], and hospital admissions and discharges [14] since 1990, as well as limited demographic [15] and vital statistics data [16]. Up to five diagnoses are recorded for each patient encounter and up to 25 diagnoses for each inpatient admission. Furthermore, it encompasses the comprehensive prescription drug database PharmaNet [17], which includes all dispensed medications for all BC residents regardless of age or funding source. Numerous general population-based studies have been successfully conducted using these databases [18–22]. In addition to these data sources, the BC Cancer registry provides data on oncology medications that are not available through PharmaNet [13]. Finally, the BC Centre for Disease Control (BCCDC) holds COVID-19 test result data (both positive and negative results) [23] and in addition, has created a Consent to Contact Registry of all individuals with a positive test who consented to be contacted for research purposes [24] We will utilize this BCCDC COVID-19 Consent to Contact Registry to contact individuals with a positive COVID-19 diagnosis for our cases, while we will use Population Data BC to contact negative and untested individuals.

### Case ascertainment and control selection

Using the administrative health databases, we identified all BC residents with a positive SARS-CoV-2 test by polymerase chain reaction (PCR), obtained via nasopharyngeal swab or saline gargle, between February 2020 and April 2021. For each COVID-19 case, we randomly selected 20 individuals from the general population who had not been tested for SARS-CoV-2 to serve as controls. To minimize the risk of misclassification bias (e.g., controls having COVID-19 but not being tested) and account for selection bias due to selective testing policies, each COVID-19 case was matched to 20 individuals from a second control group who tested negative for SARS-CoV-2. Each case was matched to both sets of controls based on age (±2 years), sex, health authority, and date of PCR test (i.e., index date ± 2 months). We used density-based sampling, meaning controls can become future cases and can be selected more than once. It is important to note that density-based sampling limits our ability to calculate the absolute risk of COVID-19 in patients with immunosuppression. Additional matching for immunosuppressive diseases (e.g, Chronic kidney disease requiring dialysis/hemodialysis) and their comorbidities that may be related to COVID-19 (e.g. heart disease, chronic lung disease, obesity etc) will be conducted using a combination of exact matching and propensity score stratification.

### Assessment of exposure

**Fig 1:**
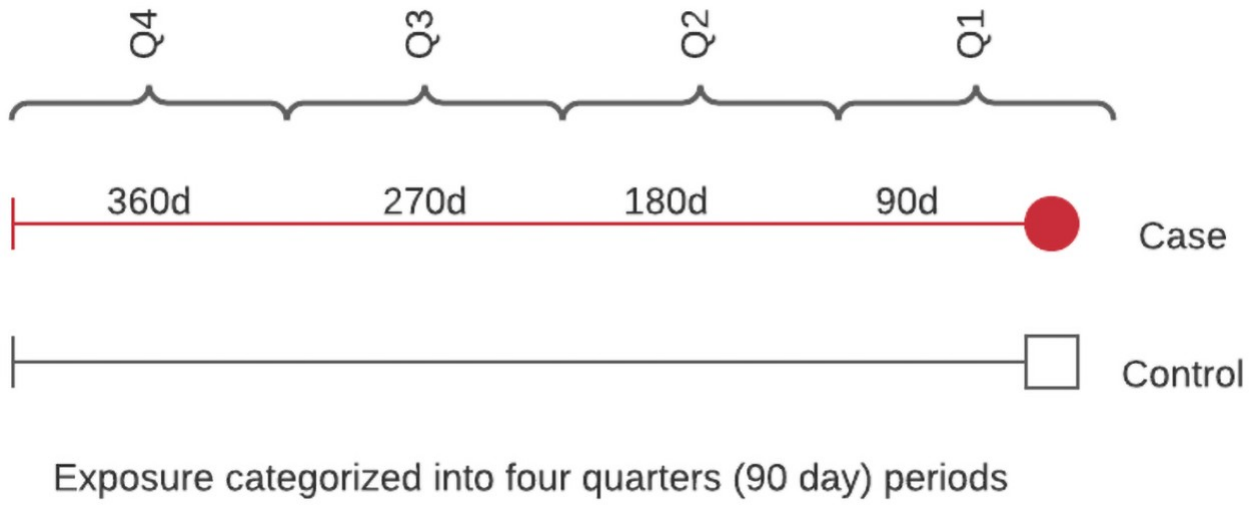
Immunomodulatory agent exposure categorized into four quarters (90-day periods).

The exposure of interest will be immunosuppressive and immunomodulatory agents used in the year prior to the index date based on prescription medication history on PharmaNet and oncology medications from the BC Cancer formulary. Drug exposure will be classified in three types: cytotoxic drugs (CD) mainly used for chemotherapy and transplants; traditional immunomodulatory agents (IA) such as hydroxychloroquine, sulfasalazine, and low dose methotrexate; and biologics and small molecule agents (e.g., rituximab, TNF, IL-6, and IL-1 inhibitors). We will assess timing of exposure to any IIA in four 90-day periods (yearly quarters) prior to index date (Fig 1). Categorizing subjects according to the recency (e.g., within the latest quarter) and regularity (e.g., number of quarters in a year) of exposure will allow us to run sensitivity analyses using alternative definitions of recent and current use. By narrowing the exposure window to the most recent quarter (i.e. 90 days prior to index) patients who received IIAs for a few days several months before the index date will not necessarily be classified as immunosuppressed. Sensitivity analyses can then be performed using larger periods of exposure (e.g., IIAs within 9 months prior to index or IIAs received for at least 2 quarters). Finally, in the case of disease-induced immunosuppression, we will include all individuals who received dialysis/hemodialysis in the 12 months preceding the index date.

### Survey sample

**Fig 2:**
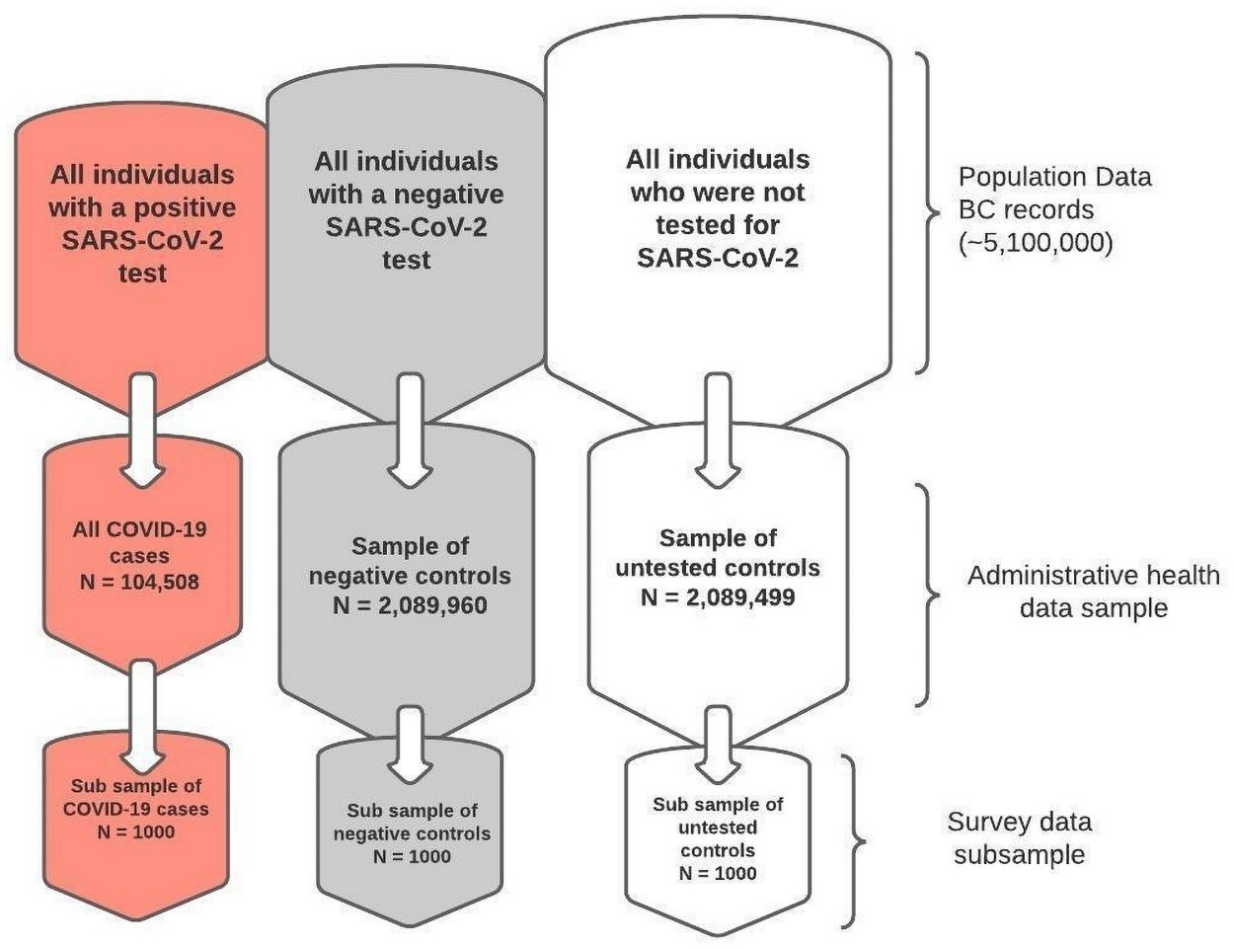
Recruitment flowchart (Records from February 2020–April 2021).

We submitted a ‘Request-to-Contact’ application to access the names and contact information of a sample of individuals from our study population for the purpose of recruiting them to our control groups for the survey. This application will be reviewed by the BC Ministry of Health’s Data Stewardship Committee and the Office of the Information and Privacy Commissioner for BC. We have used this approach in a previous study [18]. The BC Ministry of Health will select a random sample (n = 2,000; 1000 from each control group) and release their names and contact information to the research team for recruitment purposes. Additionally, we will obtain the contact information of a random sample of COVID-19 positive cases from the BCCDC registry. Together this will make our sub samples of individuals who are invited to participate in our survey (Fig 2). The random sample will be selected in the hope of capturing a generalizable cohort.

### Patient and public involvement

Engaging patients as research partners enhances the quality, relevance, and potential impact of health research [25–27]. Our research team includes four patient partners (three women, one man, all Caucasian/White) from the inflammatory arthritis, cancer, and kidney transplant communities. All patient partners had prior experience engaging in health research. The timeline for patient partner engagement is outlined in a flowchart [S1 Appendix]. To ensure effective engagement, we have enacted best practices in patient-oriented research, with support from the BC SUPPORT Unit (a provincial component of a federal program to enable health care research to be more meaningful and relevant to patients) [28]. Patient partners are collaborating in co-design and co-execution of the study, via email and videoconference (initially every 1–2 weeks then once a month), facilitated by AMH, a knowledge broker with extensive experience in patient-oriented research.

### Recruitment

The survey participants will be recruited using an adaptation of Dillman’s Total Design Method (TDM) [29]. This method has been successfully used in a similar research survey [18]. However, it was adapted to meet the Office of the Information and Privacy Commissioner regulations on the number of contacts, the timing between contact efforts and their reminders as well as the format for contact. Furthermore, the timing between contact efforts was adapted to account for potential mailing delays during the pandemic.

Potential participants will be mailed an initial package containing a personalized letter of invitation from the principal investigator (JAAZ) supplemented by a statement from our patient partners about the potential impact of the study. The package will also include a letter from the BC Ministry of Health (MoH), the consent form (with two copies of the signature page), and an addressed, prepaid envelope for returning one copy of the signed consent form. The letter of invitation will include a study description, a note on how and why we were granted access to the recipient’s name and contact information, and information regarding the protection of participant data. We will emphasize the importance of having both people with and without COVID-19 or immunosuppression participate in the study. Furthermore, the invitation letter will be tailored to be compassionate in the case that the addressee has passed away for any reason. In this case, we would request that the families of the deceased individuals fill out the survey on behalf of their deceased family member. Given the sensitive nature of possibly contacting a deceased patient’s family and the potential stigma associated with a COVID-19 diagnosis, we used the BCCDC COVID-19 language guide to ensure that all our documents were positive, inclusive, and non-stigmatizing [30].

Although financial compensations (e.g., lottery draw, cash, gift card, coupons, and money order) have been shown to increase response rates of surveys [31], our patient partners advised us to offer a donation to a charity in lieu of a raffle that would be selective (only a few people would be able to receive the incentive). As a result, the invitation package will mention that $2 will be donated to Food Banks BC for every completed survey, up to a maximum of $2,000. This form of incentive was selected as an acknowledgment, albeit nominal, of the challenges some citizens face during the pandemic.

Individuals who wish to proceed with the study will need to review and sign the consent form; one copy of the signature page will be returned to the research team in a pre-paid envelope while the other would be retained by the participant for their records. Additionally, participants will be asked to indicate their preferred survey format (online survey or mailed paper copy). A reminder letter will be mailed to potential participants who haven’t responded to the invitation package within 3 weeks. As requested by the Office of the Information and Privacy Commissioner this second contact effort will contain explicit instructions on how to withdraw from the study if the participants desire to do so. Five weeks after the initial invitation package, a third and final contact attempt will be made via phone call. Two phone calls are allowed to ensure that there were no issues with the mailing address on file or stolen mail. If the research team successfully contacts individuals via phone and they express an interest in participating in the survey another invitation package will be mailed to them at their correct address. The timeline for contacting potential participants is outlined in a flowchart [S2 Appendix].

### Data collection

Participants who opt to complete the online survey will be emailed a link to the survey hosted by Qualtrics (an online survey tool), along with a password. Participants are allowed to enter their responses in multiple sittings. The survey tool will allow the responses to be anonymized by linking them to a random study ID instead of their personally identifiable information. Those who request paper copies will be mailed the survey with a prepaid return envelope. Participants will be asked to submit the completed survey within 3 weeks after which the research team will send a survey completion reminder letter to those who have not responded.

### Survey development

The self-administered survey for this study consists of 45 questions organized into seven sections and takes approximately 20 minutes to complete [S3 Appendix]. To ensure that the survey was equivalent between the paper and online versions, both versions were pilot tested by the research staff and our patient partners at all stages. Subsequently, the research team conducted five cognitive interviews to ensure that survey questions were being interpreted similarly by participants and to determine whether questions could be modified to enhance comprehension and reduce respondent burden. Cognitive interview participants were recruited via email from the Principal Investigator’s networks. Participants for the cognitive interviews were selected because they had tested positive for SARS-CoV-2 or had some form of immunosuppression.

In preparation for these cognitive interviews, the research team created an interview guide with general cognitive probes and item-specific questions [S4 Appendix]. The 60–90 minute interviews were held over Zoom and were led by MA or SM with the support of ZF or JK. Following these interviews, the research team discussed and agreed by consensus which items to keep, modify or remove. Respondent feedback regarding logical errors, ambiguity or outdated terminology was incorporated in the final form of the survey. However, questions that had been previously used in large surveys or standardized questionnaires were left in their original form.

### Survey components

→ Section One: Questions from previously used health questionnaires will be used to collect data on sociodemographic variables (e.g., gender identity, marital status, cultural background, household income, and educational attainment) and clinical variables (e.g., age, height, weight, pregnancy) [32–34].
→ Section Two: While the relationship between smoking and COVID-19 is still unclear, the increased hand-mouth contact, lung damage, and associated comorbidities may increase the risk of infection and severe outcomes of COVID-19 [35]. We will use questions from the 2019 and 2020 Canadian Community Health Surveys to ask respondents to estimate how often they used vaping devices or smoked cigarettes, cannabis, or other tobacco products [36, 37]. Respondents will also be asked if anyone in their household smokes cigarettes daily to account for the health effects of second-hand smoke.
→ Section Three: We will collect data on participants’ vaccination history including seasonal flu shots and the vaccines recommended by the BC immunization schedules [37, 38]. These questions will be used as a proxy for health consciousness and willingness to engage in health promoting behaviours outside a pandemic context.
→ Section Four: Questions on occupation, mode of transportation and workplace contact with the public were largely based on the BCCDC COVID-19 SPEAK survey [39–41]. Respondents will be asked about their occupational status during Phase 1 of BC’s Restart Plan (March-May 2020) when provincial protective measures were introduced, and again during Phases 2 and 3 (June 2020- present) when guidelines changed.
→ Section Five: Using questions from the BCCDC COVID-19 SPEAK survey, we will ask participants how often they followed certain precautions to reduce their likelihood of exposure to SARS-CoV-2 virus (e.g., frequent hand washing and social distancing). We will also collect data on face mask usage, travel history, COVID-19 testing, and visits to medical facilities [33].
→ Section Six: This section uses a Likert-type scale to inquire about participants’ perceived vulnerability (or resilience) to the SARS-CoV-2 virus. Specifically, it will ask participants to what extent they agree or disagree with certain statements (e.g., I feel that I am more vulnerable to COVID-19 because of a medical condition).
→ Section Seven: In the last section, participants will be asked if they would be interested in receiving information about results of this study and whether they would like to receive information about future studies. Participants were also given the opportunity to leave feedback or provide additional details.

### Data analysis plan

We compared the baseline characteristics between cases and matched control using proc means procedure in SAS for continuous variables and proc frequency procedure in SAS for binary/categorical variables. A conditional logistic regression model will be constructed to adjust for covariates and possible confounders (e.g., comorbidities and survey covariates) to examine the odds ratio of COVID-19 infection among immunosuppressed individuals. Missing values for confounders in BC survey data due to nonresponses will be addressed using our multiple imputation procedure [42], which imputes missing values under the standard assumption of data missing at random (MAR), but does not require imposing parametric distributional assumptions for missing data, and can accommodate nonlinear and interactive relationships among variables. All statistical analyses were conducted using SAS version 9.4 (SAS Institute Inc).

The sample size for is based on ~3,600 COVID-19 positive cases identified by Jun 30th 2020 (conservative rate of 25 cases per day for May and June) and up to 72K controls (min 14.4K, max 36K for each control group) with ≥ 5% of controls and 8% cases exposed to IIAs in the last 12 months prior to index date; we will have 90% power to detect a minimum odds ratio of 1.3. Under the assumption that 8% of 3600 COVID positive cases were exposed to immunosuppressive or immunomodulators, we will have 80% power to detect a minimum hazard ratio of 0.85 for decreased risk of hospitalization, admission to intensive care unit and mortality for cases exposed to immunosuppressive or immunomodulators.

### Privacy & confidentiality

We have implemented measures to keep all personal information secure, including names, contact information, survey data, and medical reports. These will be kept in secure locations accessible only to a restricted number of study personnel at Arthritis Research Canada. Participants will only be identified by their study ID; meaning that their personally identifiable information (i.e., name, address, or phone number) will never be linked to their survey responses nor administrative health data (e.g., SARS-CoV-2 test results).

We also enacted measures to ensure that participants’ disease status (e.g., COVID-19 or immunosuppression) would not be accidentally disclosed to the research team or other individuals during study recruitment. For example, the invitation letter was written in a way that communicates that the individual in question was randomly selected to participate in the study as someone who ‘may or may not have been tested for SARS-CoV-2’ and ‘may or may not have immunosuppression’. In cases where the research team must leave a voicemail when attempting to contact participants, they will avoid mentioning any specific diagnoses and only identify the study as a ‘health research study conducted at the University of British Columbia’. Finally, all data collected from the surveys will be uploaded to Population Data BC’s Secure Research Environment (SRE) where it will be linked to a study ID and analyzed securely.

## Results to date

Survey subsample has not been selected yet and therefore we present preliminary data on the entire study population (i.e., all COVID-19 cases and a sample of each control group). The administrative data underlying this article were accessed from Population Data BC, https://www.popdata.bc.ca/data.

### Baseline characteristics

**Fig 3:**
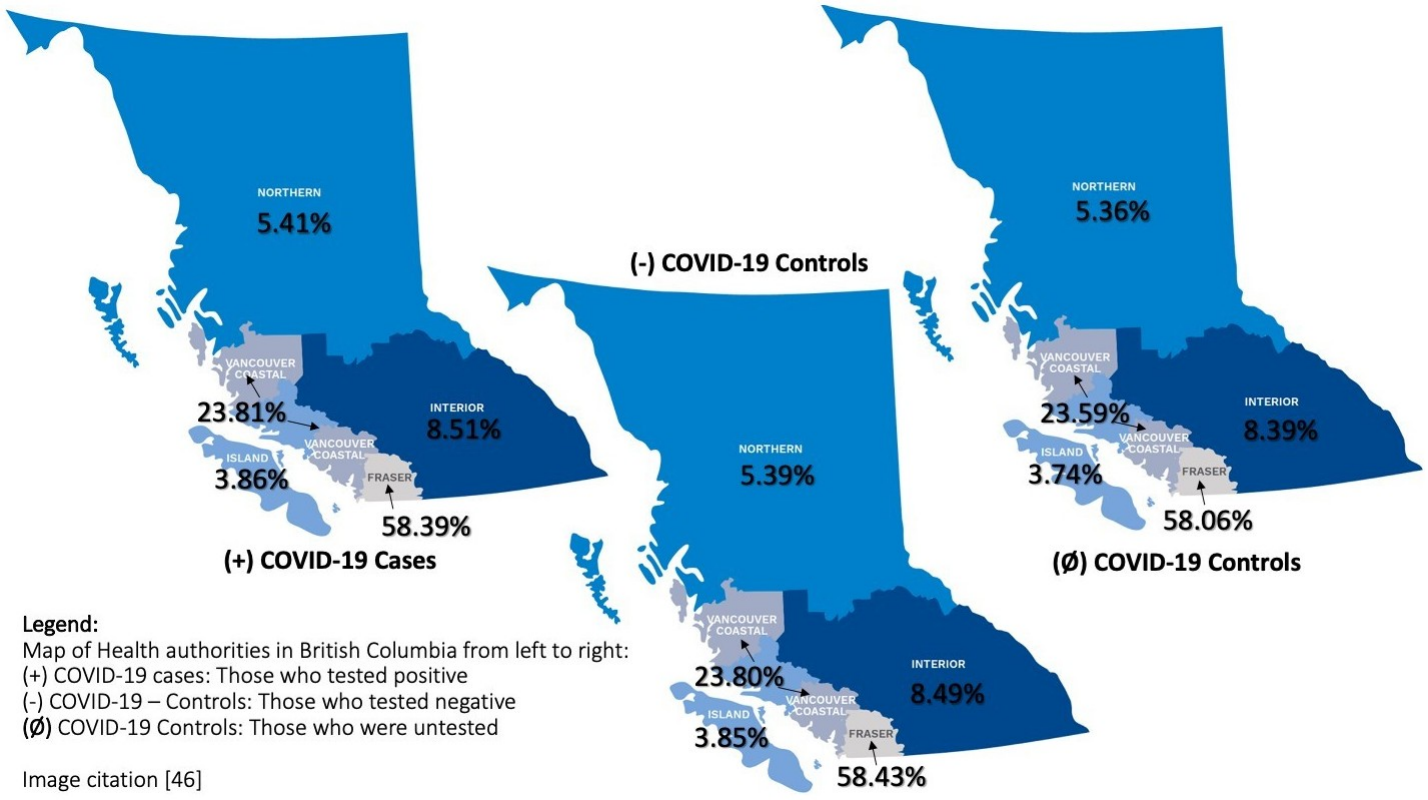
Geographic distribution of COVID-19 cases and controls. Image citation [46].

We used administrative health databases to identify 104,508 laboratory-confirmed cases of COVID-19, 2,089,960 negative controls, and 2,089,499 untested controls from BC’s five health authorities. Baseline characteristics, comorbidities and medication use are presented in Tables 1–3 and geographic distribution of cases and controls is presented in Fig 3. This information is current as of April 11th, 2021, since then more than 40,000 additional cases have been identified [43]. Differences between case and two control groups and interactions between variables (e.g., sex X immunosupressive drug, comorbidities etc.) will be analyzed after data collection is completed and presented in future publications.

**Table 1:** Baseline characteristics of COVID-19 cases, negative controls, and untested controls.

Variable	COVID-19 Cases (n = 104,508)	Negative Controls (n = 2,089,960)	Untested Controls (n = 2,089,499)
**Mean age (SD)**	39.85 (20.09)	39.85 (20.07)	39.82 (20.09)
**% Female**	49.09	49.09	49.09
**Rural residence (%)**	5.08	6.03	6.58
**Charlson comorbidity index score, mean (SD)**	0.30 (1.00)	0.36 (1.12)*	0.18 (0.72)*
**Mean number of hospitalizations** ^1^ **(SD)**	0.17 (0.66)	0.22 (0.77)*	0.09 (0.43)*

^1^ Hospitalization per person in the year prior to index date

* p < 0.0001

**Table 2:** Comorbidities in the year prior to index date for COVID-19 cases, negative controls, and untested controls.

Comorbidities, N (%)	COVID-19 Cases	Negative Controls		Untested Controls	
	(n = 104,508)	(N = 2,089,960)	P-value	(N = 2,089,499)	P-value
Chronic obstructive pulmonary disease	5,351 (5.1)	141,642 (6.8)	<.0001	71,909 (3.4)	<.0001
Asthma	3,549 (3.4)	89,640 (4.3)	<.0001	47,303 (2.3)	<.0001
Angina	1,079 (1.0)	26,676 (1.3)	<.0001	13,305 (0.6)	<.0001
Obesity*	614 (0.6)	151,178 (0.7)	<.0001	8,946 (0.4)	<.0001
Surgery	164 (0.2)	4,585 (0.2)	<.0001	2,455 (0.1)	0.0003
Liver Disease related to alcohol	1,043 (1.0)	22,856 (1.1)	0.0037	7,522 (0.4)	<.0001
Myocardial Infarction	3,174 (3.0)	76,531 (3.7)	<.0001	42,945 (2.1)	<.0001
Stroke	1,295 (1.2)	29,674 (1.4)	<.0001	13,704 (0.7)	<.0001
Peripheral vascular disease	620 (0.6)	17,261 (0.8)	<.0001	9,095 (0.4)	<.0001
Congestive Heart Failure	1,618 (1.6)	41,449 (2.0)	<.0001	17,242 (0.8)	<.0001
Hypertension	12,256 (11.7)	239,451 (11.5)	0.0075	196,247 (9.4)	<.0001
Chronic Kidney Disease	1,831 (1.8)	42,224 (2.0)	<.0001	24,651 (1.2)	<.0001
Inflammatory Bowel Disease	334 (0.3)	9,837 (0.5)	<.0001	5,110 (0.2)	<.0001
Cancer	6,864 (6.6)	186,727 (8.9)	<.0001	122,279 (5.9)	<.0001
Sepsis	877 (0.8)	22,909 (1.1)	<.0001	3,411 (0.2)	<.0001
Varicose veins	609 (0.6)	11,447 (0.6)	0.135	7,848 (0.4)	<.0001
Trauma	335 (0.3)	8,875 (0.4)	<.0001	3,481 (0.2)	<.0001
Fractures	1,115 (1.1)	25,971 (1.2)	<.0001	12,708 (0.6)	<.0001

All comorbidities were identified by ICD-9 code (* Obesity only includes those who sought medical attention for the condition).

**Table 3:** Medication used in the year prior to index date for COVID-19 cases, negative controls, and untested controls.

Medications, N (%)	COVID-19 Cases	Negative Controls		Untested Controls	
	(n = 104,508)	(N = 2,089,960)	P-value	(N = 2,089,499)	P-value
Glucocorticoids	3,848 (3.7)	98,696 (4.7)	<.0001	49,121 (2.4)	<.0001
Cardiovascular medications	7,604 (7.3)	166,349 (8.0)	<.0001	113,920 (5.5)	<.0001
Fibrates	88 (0.1)	2,085 (0.1)	0.1186	1,548 (0.1)	0.2422
Anti-diabetic medications	3,712 (3.6)	55,112 (2.6)	<.0001	43,156 (2.1)	<.0001
NSAIDs*	6,711 (6.4)	111,435 (5.3)	<.0001	73,767 (3.5)	<.0001
Aspirin*	590 (0.6)	10,315 (0.5)	0.0014	4,676 (0.2)	<.0001
Statin	951 (0.9)	17,861 (0.9)	0.0581	17,030 (0.8)	0.0009

* Only includes medications dispensed by a pharmacist.

### Next steps

**Fig 4:**
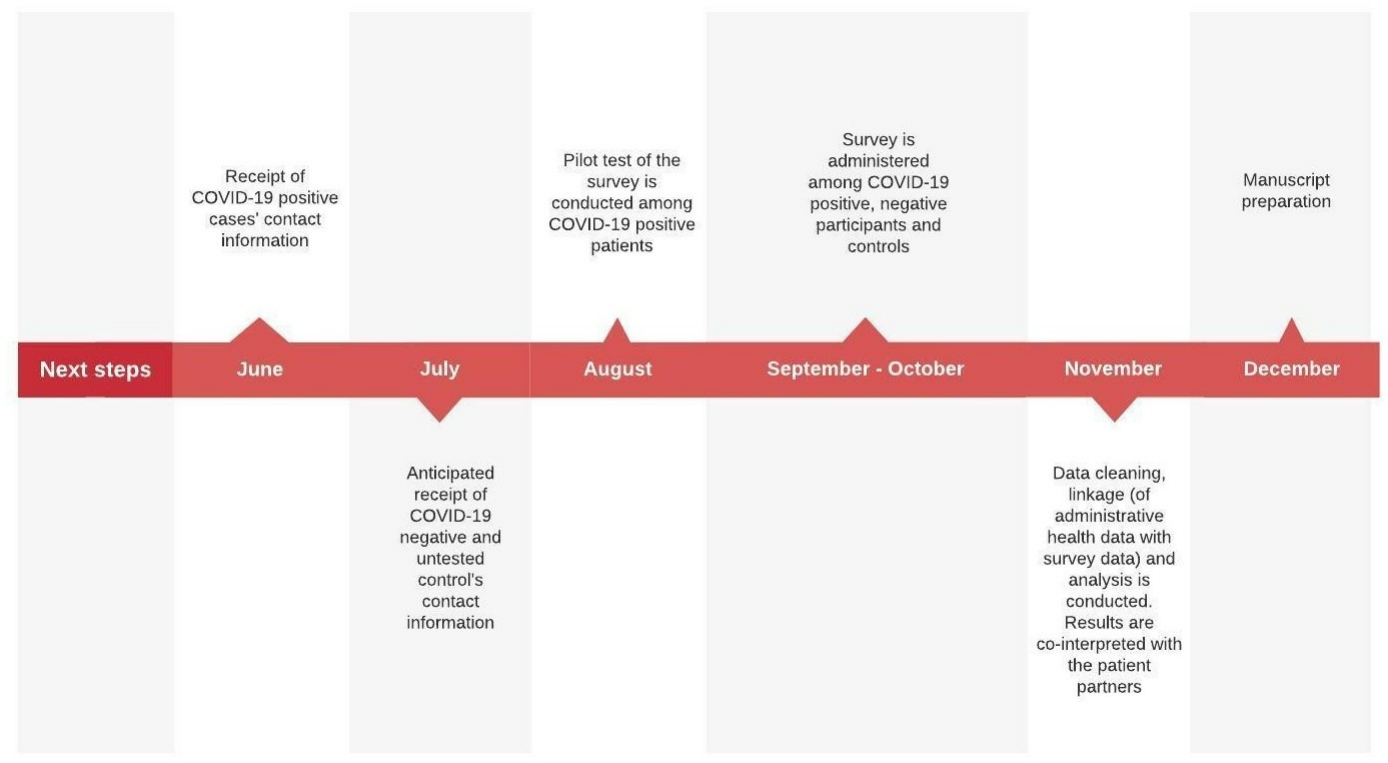
Anticipated timeline of project completion.

As of August 2021, we have sent out recruitment invitations to collect survey data from the first 500 participants and anticipate the completion of recruitment by the end of the year. The timeline of our study is outlined in Fig 4.

### Strengths and limitations

One strength of our study is the selection of participants with and without COVID-19 which is from a population-based sample of 5.1 million people, allowing our results to be generalizable at the population level. By recruiting a random sample of these individuals to participate in our survey, we aimed to minimize selection bias and establish a representative cohort with different sociodemographic variables, immune status, and health preventative behaviours. Nevertheless, the representativeness of our survey cohort will ultimately depend on the individuals who consent to participate and complete our survey. We are further limited by the representativeness of the BCCDC COVID-19 Consent to Contact Registry. If those who agree to participate differ in their susceptibility or exposure to COVID-19 and preventive behaviours from those who decline to participate, the generalizability of our findings will be reduced.

Another concern is our ability to recruit individuals who do not have a COVID-19 diagnosis or any form of immunosuppression, since they will not have the same motivation to participate as those with either diagnosis. Additionally, individuals who tested positive for the SARS-Cov-2 virus have been invited to participate in many research projects since the start of the pandemic. The resulting respondent fatigue may impact their decision to complete our survey. To combat this, we are making efforts to ensure a broad range of individuals chose to complete our survey. For example, we are providing the option of paper surveys to encourage those without access to a computer or those who are less comfortable using them, and an online version for individuals who find it safer or more convenient. Participants can complete the survey at their own pace and don’t have to participate in in-person visits or telephone interviews. This may be preferable for those with unpredictable schedules, family responsibilities or health issues that would hinder their ability to participate. Finally, in cases where an individual has passed away from complications of COVID-19, we invite their loved ones to complete the survey on their behalf in an effort to recruit individuals across a range of COVID-19 severities.

While the survey allows us to collect data on clinical and behavioral variables, the validity of these self-reported data may be subject to bias. Nevertheless, the validity of self-reported body weight data has been supported in previous Canadian research [44]. Even though the questionnaires we used are generic instruments [32, 33, 36, 37, 41] or have been used in previous projects [18, 34], we acknowledge that they have not been formally validated for populations with COVID-19. At the time this survey was developed there were no validated COVID-19 questionnaires in use due to the novelty of this virus. However, the data gathered by the BCCDC COVID-19 SPEAK survey has been used by policymakers and community stakeholders to inform public health guidance [45]. Another limitation of our study is the accuracy and completeness of medication profiles found in PharmaNet (electronic provincial medication database). For example, NSAID use will only be captured by PharmaNet with dispensed by a pharmacist and therefore will miss any instances where it was purchased over-the-counter. Moreover, PharmaNet only captures the medication dispensed which may not capture the dose and frequency discrepancies due to imperfect adherence. Nevertheless, PharmaNet remains a comprehensive prescription drug database and has been successfully used to conduct various population-based studies [18–20, 22].

Despite the methodological limitations, to the best of our knowledge, this will be the first health research project in Canada to link administrative health data, laboratory data and self-reported survey data allowing us to assess the risk of COVID-19 in patients with various forms of immunosuppression.

## Supporting information

S1 Appendix Timeline for patient partner engagement. (PNG)

S2 Appendix Timeline for contacting potential participants. (PDF)

S3 Appendix BC-COVID-19 survey. (PDF)

S4 Appendix Cognitive interviewing guide. (PDF)
