# Supplementary figures and images for: Immunosuppression and COVID-19 infection in British Columbia: Protocol for a linkage study of population-based administrative and self-reported survey data
Source: PLoS One. 2021 Nov 19;16(11):e0259601. doi: 10.1371/journal.pone.0259601 (PMC8604283; doi:10.1371/journal.pone.0259601)

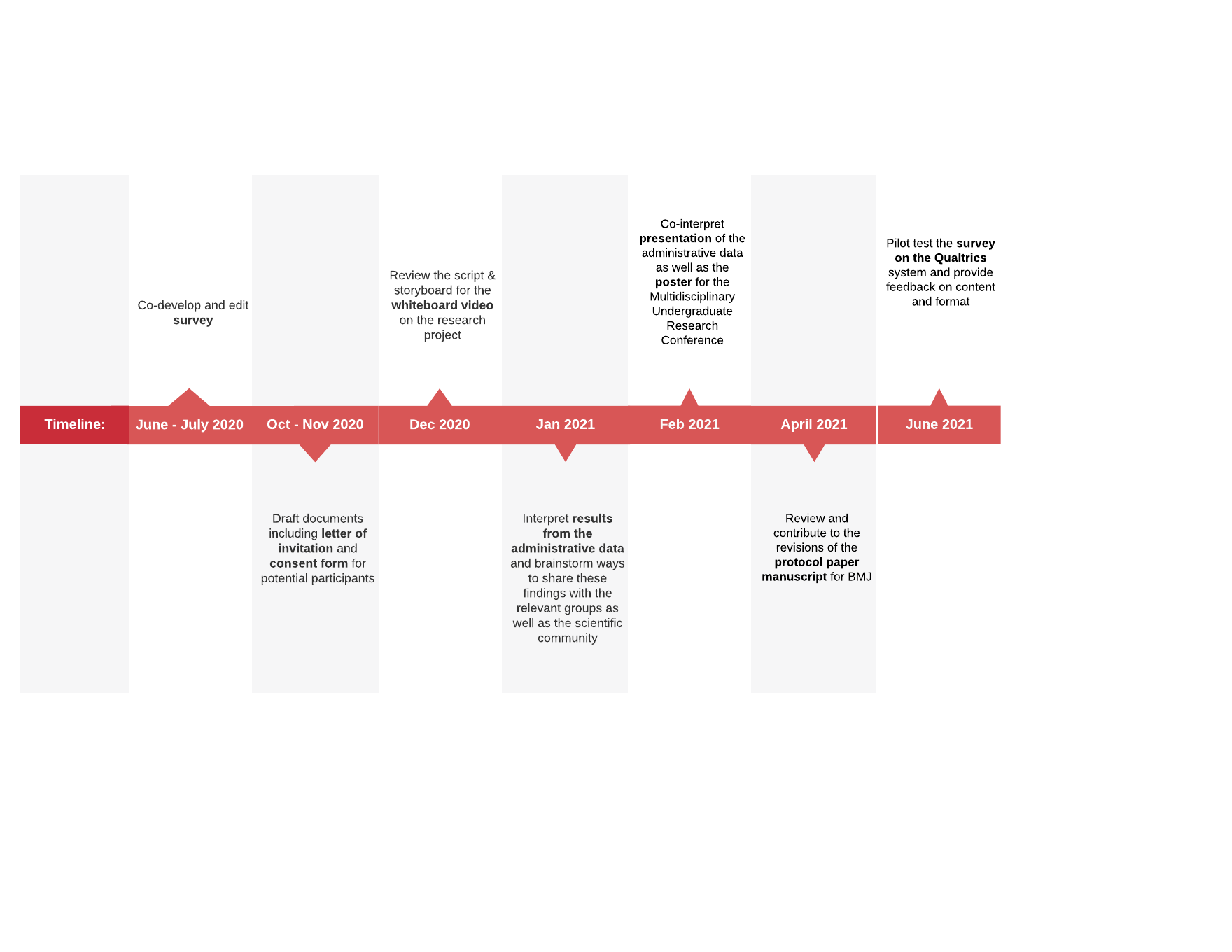

Supplement: S1 Appendix — (PNG) [file pone.0259601.s001.png]

## BC COVID-19 Survey Timeline

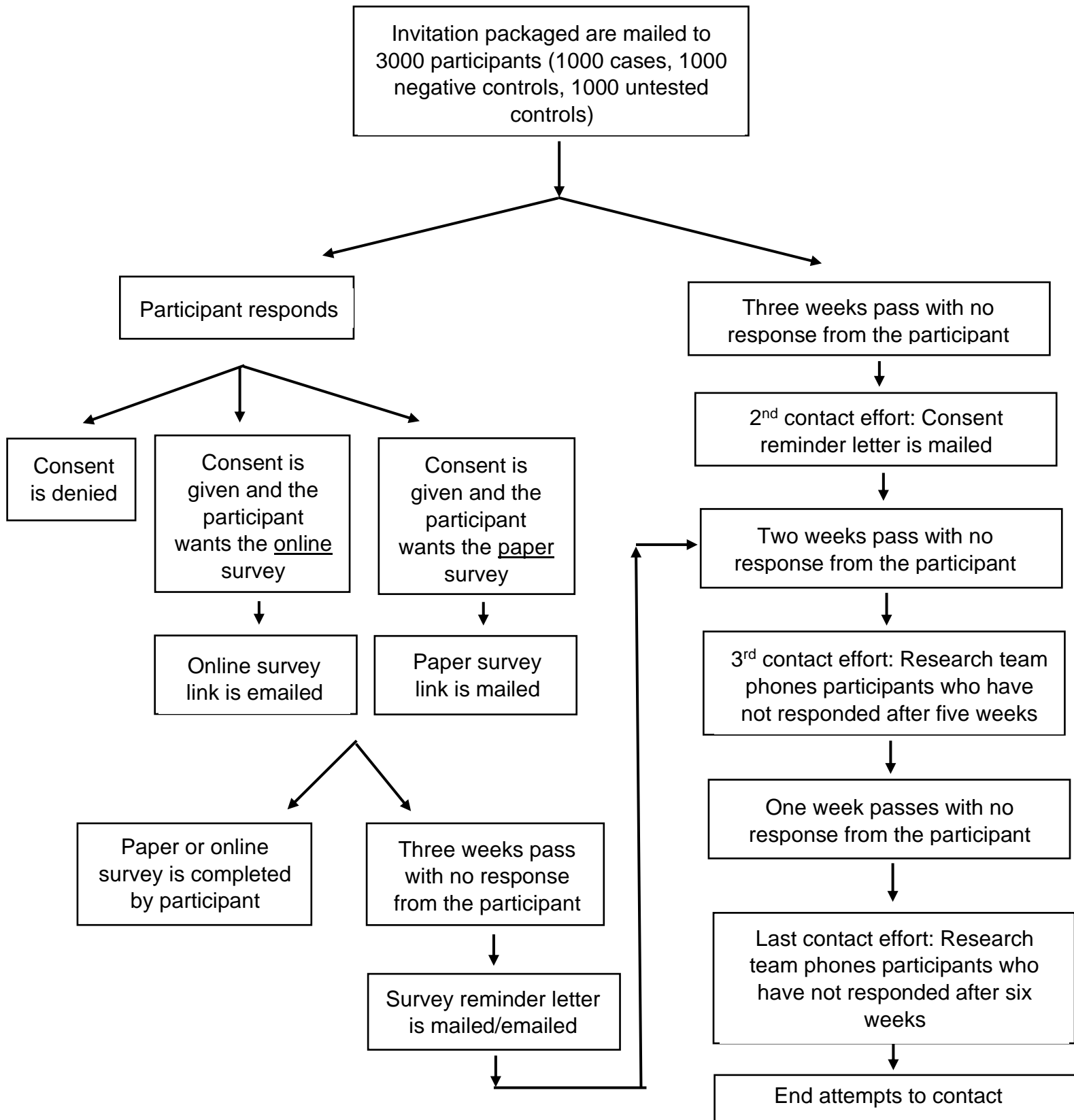

Supplement: S2 Appendix — (PDF) [file pone.0259601.s002.pdf]
