# Supplementary material for: Immunosuppression and COVID-19 infection in British Columbia: Protocol for a linkage study of population-based administrative and self-reported survey data
Source: PLoS One. 2021 Nov 19;16(11):e0259601. doi: 10.1371/journal.pone.0259601 (PMC8604283; doi:10.1371/journal.pone.0259601)
Supplement: S3 Appendix — (PDF) [file pone.0259601.s003.pdf]

Participant ID: ________________

COVID-19 – Survey of the BC Population

Survey of People with and Without Immune Suppression

Dear participant:

Thank you for your interest in this survey. Our research is exploring the link between being immune-compromised (people living with a weak immune system) and COVID-19. The questions in this survey will ask you about your background, health, lifestyle, and habits during the COVID-19 pandemic.

Your answers are **essential** to the success of this study – whether or not you have had a test for COVID-19. Your participation in this research will help British Columbians who are immune-compromised.

The survey has seven parts and should take **about 20 minutes to complete** (<u>you may do it over many sessions within a total of 30 days</u>).

For each question, please choose (☑) the answer to the question that best 'fits' you. If a question specifies "please check (✓) all that apply", then you may choose more than one answer.

For questions with boxes, please fill in the boxes with numbers like this:

2	1
**Weeks**	

Please remember, all information you provide will be kept **confidential** – your answers will be combined with those from others so that no answers can be traced specifically to you. **You do not have to answer any questions you do not wish to**.

If you have any questions or concerns about this study, please call 604-207-4040, or toll-free 1-855-907-0400, or e-mail covid.survey@arthritisresearch.ca. Collect calls are accepted.

Once again, thank you for participating in this study.

Section 1: Your Background

In this section, we are interested in learning a little bit about your background. We know that social and economic ($) factors can affect people's health in both negative and positive ways, and sometimes their impact may not be obvious. We are interested in learning how social and economic factors may affect the likelihood of getting COVID-19 and if the symptoms may be worse.

1. **What is your year of birth?**

**Year**			

2. **What is your height?**

**Ft**	**In**	

or

**cm**		

3. **What is your weight?**

**lbs**		

or

**kg**		

4. **Differences in sex and gender affect our health. Sex (being born male or female) and gender (social and cultural factors) can affect things like your risk of developing certain diseases, response to medical treatments, and how often you seek health care.**

   What was your sex at birth?

   ☐ Male
   ☐ Female
   ☐ Intersex
   ☐ I prefer not to answer

5. **What is your current gender identity?**
   ☐ Male
   ☐ Female
   ☐ Transgender male / trans man
   ☐ Transgender female / trans woman
   ☐ Non-binary, genderqueer, genderfluid
   ☐ Indigenous or cultural gender minority (e.g. two-spirit)
   ☐ Other: ________________________________
   ☐ I prefer not to answer

6. **What is the highest level of education you have completed? (Please check (✓) only one):**
   ☐ Elementary school
   ☐ Some high school
   ☐ High school completed
   ☐ Technical/trade/vocational college
   ☐ Community college
   ☐ Some university/college
   ☐ University degree obtained
   ☐ Graduate or post-graduate training
   ☐ I prefer not to answer

7. **What is your current marital status?**
   ☐ Married/engaged
   ☐ Long-term or common-law relationship
   ☐ Widowed
   ☐ Separated or divorced
   ☐ Single, never married
   ☐ I prefer not to answer

8. **How many people who currently live in your household are in the following age categories? Please include yourself if applicable.**

Children	Adults	Seniors
[ ][ ]	[ ][ ]	[ ][ ]
**Aged 18 years or younger**	**Aged 19 – 64 years**	**Aged 65 years or older**

9. Are you currently pregnant?

☐ No → Skip to question 11
☐ Not applicable (biologically male, unable to conceive, or other reason) → Skip to question 11
☐ I don't know → Skip to question 11
☐ Yes → Continue to question 10

10. If yes, how many weeks along are you?

**Weeks**	

11. People living in BC come from many different cultural and racial backgrounds. (Please check (✓) all that apply to you):

☐ Indigenous/Aboriginal, Inuit, Métis
☐ White
☐ Latino/Hispanic
☐ African, Caribbean, or Black
☐ West Asian (e.g., Iranian, Afghan, Iraqi)
☐ Arab
☐ South Asian (e.g., Indian, Pakistani, Sri Lankan, Bangladeshi)
☐ Southeast Asian (e.g., Vietnamese, Cambodian, Malaysian, Laotian)
☐ Chinese
☐ Korean
☐ Japanese
☐ Filipino
☐ Other (please specify): _________________
☐ I don't know
☐ I prefer not to answer

12. For some people, spirituality is religion, and for others it might mean something different. Using a definition of spirituality that is meaningful to you, do you have a regular spiritual practice?

☐ Yes
☐ No
☐ I prefer not to answer

13. Studies have found that income level can affect your health. What is your best estimate of the total income for all members of your household (from <u>all sources</u>, before taxes and deductions) in the past 12 months? Examples include income from jobs, freelance work, rental income, investments, pensions, and scholarships.

☐ $0.00-$19,999
☐ $20,000-$39,999
☐ $40,000-$59,999
☐ $60,000-$79,999
☐ $80,000-$99,999
☐ $100,000-$149,999
☐ $150,000-$199,999
☐ $200,000 and over
☐ I don't know
☐ I prefer not to answer

Section 2: Smoking

In this section we are interested in learning more about your smoking habits, including the use of cigarettes, vaping devices, and other tobacco or cannabis products. The information is relevant because COVID-19 can cause lung problems.

14. **Does someone in your household, other than yourself, smoke cigarettes daily?**
   ☐ Yes
   ☐ No
   ☐ I don't know

15. **Do you currently use or have you ever used any tobacco products? Examples include cigarettes, vaping devices, e-cigarettes, and chewing tobacco.**
   ☐ No → Skip to question 21
   ☐ Yes, either currently or in the past → Continue to question 16

16. **At the present time, do you smoke cigarettes? Please exclude e-cigarettes, vaping devices, and other tobacco products such as cigars, cigarillos, water pipes, and chewing tobacco.**
   ☐ Not at all → Skip to question 18
   ☐ Occasionally → Continue to question 17
   ☐ Daily → Continue to question 17

17. **On the days that you do smoke, about how many cigarettes do you usually smoke?**

**Cigarettes**	

18. **Do you use any of the following tobacco products at least once a week (please check (✓) all that apply):**
   ☐ Little cigars or cigarillos
   ☐ Cigars
   ☐ Tobacco smoked in a traditional pipe
   ☐ Chewing tobacco, pinch, or snuff
   ☐ Tobacco water-pipe
   ☐ Other tobacco products: ________________________
   ☐ None of the above

19. In the past 30 days, how often did you use an electronic cigarette (e-cigarette) or vaping device? Please exclude vaping cannabis as this will be asked in a later question.

☐ Daily

☐ Less than once a day, but at least once a week

☐ Less than once a week, but at least once in the past month

☐ Not at all

20. If you currently do not smoke or use tobacco products, have you ever smoked or used tobacco products (including e-cigarettes and vaping devices) daily in the past? Please do not include cannabis products as this will be asked in a later question.

☐ Yes

☐ No

☐ I prefer not to answer

21. How often did you smoke or vape cannabis in the past 12 months? Please do not include any cannabis products that are ingested or applied to the skin.

☐ Not at all

☐ Less than once a month

☐ 1-3 times a month

☐ Once a week

☐ More than once a week

☐ Daily or almost daily

☐ I prefer not to answer this question

Section 3: Vaccinations

The next set of questions are about your vaccination history. Different vaccines are recommended by the Government of Canada depending on a person's age, occupation, and health status.

22. **Have you ever had a seasonal flu shot?**
    ☐ No → Skip to question 24
    ☐ I don't know → Skip to question 24
    ☐ Yes → Continue to question 23

23. **When did you have your last seasonal flu shot?**
    ☐ This flu season
    ☐ Last flu season
    ☐ Two or more flu seasons ago
    ☐ I don't know

24. **Which of the following vaccines have you ever received? (Please check (✓) all that apply)**:

**Vaccinations that are recommended to the general public in British Columbia**	**Check (✓) if definitely yes**	**Check (✓) if likely yes**	**Check (✓) if definitely no**
Tetanus-Diphtheria-Pertussis (Tdap)	☐	☐	☐
Measles-Mumps-Rubella (MMR)	☐	☐	☐
Meningococcal disease	☐	☐	☐
Pneumococcal disease	☐	☐	☐
Hepatitis B	☐	☐	☐
Polio	☐	☐	☐
Haemophilus influenzae type b (a bacterial disease, not influenza (flu))	☐	☐	☐
Rotavirus	☐	☐	☐
Chickenpox (BC began vaccination for chickenpox in 2005)	☐	☐	☐

Human Papilloma Virus (HPV)	☐	☐	☐
H1N1 (swine flu; vaccination occurred in 2009 – 2010 during the H1N1 pandemic)	☐	☐	☐
COVID-19	☐	☐	☐
**Vaccinations that are recommended based on occupation, travel, age, or health status**			
Hepatitis A	☐	☐	☐
Tuberculosis (BCG)	☐	☐	☐
Rabies	☐	☐	☐
Smallpox	☐	☐	☐
Cholera	☐	☐	☐
Japanese encephalitis	☐	☐	☐
Tick-borne encephalitis	☐	☐	☐
Typhoid	☐	☐	☐
Dengue	☐	☐	☐
Yellow fever	☐	☐	☐
Shingles	☐	☐	☐

Section 4: Occupation

In this section, we are interested in learning about your employment during the COVID-19 pandemic. A person's place of employment or transportation to their place of employment may increase the likelihood of getting COVID-19 or other viruses.

We will be asking questions about your employment since British Columbia began strict physical distancing measures (March 2020 – May 2020, also known as the first wave of the pandemic) and since certain restrictions have been lifted (June 2020 – present). If you have more than one job, please choose the one where you work the most hours.

Phase 1 (March 2020 – May 2020): Strict physical distancing

25. **Please pick the option which best describes your situation during March 2020 – May 2020. If your working status changed in this time period, please choose the option that describes your situation during most of this time period.**

☐ In full- or part-time education
☐ Unemployed due to COVID-19
☐ Unemployed for reasons other than COVID-19
☐ Unable to work for health reasons
☐ Unable to work for non-health reasons
☐ Looking after my home/family
☐ Retired from paid work
☐ On maternity/paternity leave
☐ Other: ________________________________

(All of the above:) Skip to question 30

☐ Employed full time (including self-employed or on a work training program) → Continue to question 26
☐ Employed part time (including self-employed or on a work training program) → Continue to question 26

26. **Which best describes your work situation from March 2020 – May 2020? If your working status changed in this time period, please choose the option that describes your situation during most of this time period.**

☐ I continued to go out of my home to work
☐ I worked from home due to the COVID19 pandemic; but previously used to work out of my home
☐ I worked from home even before the COVID19 pandemic

27. From <u>March 2020 – May 2020</u>, how many members of the public were you in direct contact with at your place of occupation, not including coworkers?

☐ 0 people
☐ 1 – 5 people
☐ 6 – 10 people
☐ 11 – 50 people
☐ More than 50 people

28. What industry did you work in from <u>March 2020 – May 2020</u>?

☐ Accommodation and food services
☐ Administrative and support, waste management, and remediation services
☐ Agriculture, forestry, fishing, and hunting
☐ Arts, entertainment, and recreation
☐ Construction
☐ Educational services
☐ Finance and insurance
☐ Health care
☐ Information and cultural industries
☐ Management of companies and enterprises
☐ Manufacturing
☐ Mining, quarrying, and oil and gas extraction
☐ Professional, scientific, and technical services
☐ Real estate and rental and leasing
☐ Retail trade
☐ Social assistance (e.g., social worker)
☐ Transportation and warehousing
☐ Utilities
☐ Wholesale trade
☐ Other: ______________________________
☐ I prefer not to answer

29. If you had a job that is considered an essential service from <u>March 2020 – May 2020</u>, please specify what type:

☐ Health care worker (e.g., nurse, physician, physiotherapist, pharmacist)
☐ Grocery store worker
☐ Transportation (e.g., public transit, taxi, trucking, piloting)
☐ Law enforcement
☐ Construction/trades
☐ Other: _____________________________
☐ I did not have a job that was considered an essential service

30. Which of the following types of transportation did you use at least once per week, from <u>March 2020 – May 2020</u>? Please include transportation to work, school, medical appointments, and other appointments (please check (✓) all that apply):

☐ Walking or running
☐ Bicycling
☐ Public transit (bus, SkyTrain, train)
☐ Drive alone
☐ Drive with one or more people
☐ Stayed at home
☐ Other: ____

Phase 2 & 3 (June 2020 – present): Moderate physical distancing

31. **Please pick the option which best describes your situation during <u>June 2020 – present</u>. If your working status changed in this time period, please choose the option that describes your situation during most of this time period.**
    - ☐ In full- or part-time education
    - ☐ Unemployed due to COVID-19
    - ☐ Unemployed for reasons other than COVID-19
    - ☐ Unable to work for health reasons
    - ☐ Unable to work for non-health reasons
    - ☐ Looking after my home/family
    - ☐ Retired from paid work
    - ☐ On maternity/paternity leave
    - ☐ Other: ______________________________

    (all of the above) → Skip to question 35
    - ☐ Employed full time (including self-employed or on a work training program) → Continue to question 32
    - ☐ Employed part time (including self-employed or on a work training program) → Continue to question 32

32. **Which best describes your work situation from <u>June 2020 – present</u>? If your working status changed in this time period, please choose the option that describes your situation during most of this time period.**
    - ☐ I continued to go out of my home to work
    - ☐ I worked from home due to the COVID19 pandemic; but previously used to work out of my home
    - ☐ I worked from home even before the COVID19 pandemic and continued to do so

33. **From <u>June 2020 – present</u>, how many members of the public were you in direct contact with at your place of occupation, not including coworkers?**
    - ☐ 0 people
    - ☐ 1 – 5 people
    - ☐ 6 – 10 people
    - ☐ 11 – 50 people
    - ☐ More than 50 people

34. What industry did you work in from <u>June 2020 – present</u>?

☐ Accommodation and food services
☐ Administrative and support, waste management, and remediation services
☐ Agriculture, forestry, fishing, and hunting
☐ Arts, entertainment, and recreation
☐ Construction
☐ Educational services
☐ Finance and insurance
☐ Health care
☐ Information and cultural industries
☐ Management of companies and enterprises
☐ Manufacturing
☐ Mining, quarrying, and oil and gas extraction
☐ Professional, scientific, and technical services
☐ Real estate and rental and leasing
☐ Retail trade
☐ Social assistance (e.g., social worker)
☐ Transportation and warehousing
☐ Utilities
☐ Wholesale trade
☐ Other: ________________________________
☐ I prefer not to answer

35. Which of the following types of transportation did you use at least once per week, from <u>June 2020 – present</u>? Please include transportation to work, school, medical appointments, and other appointments (please check (✓) all that apply):

☐ Walking or running
☐ Bicycling
☐ Public transit (bus, SkyTrain, train)
☐ Drive alone
☐ Drive with one or more people
☐ Stayed at home
☐ Other: ____

Section 5: COVID-19 Prevention

In this section, we would like to know the things that you may or may not have done that have been recommended by health officials regarding COVID-19. Your answers will be combined with that from others, so that no answers can be traced specifically to you. <u>If you tested positive for COVID-19, please specify which things you were doing before you tested positive.</u>

36. Which of the following precautions have you taken to reduce your likelihood of exposure to COVID-19 (please check (✓) all that apply):

	Never	Rarely	Often	Very frequently
I wash my hands for at least 20 seconds at a time	☐	☐	☐	☐
I avoid touching my eyes, nose, and mouth with unwashed hands	☐	☐	☐	☐
I use disinfectants to clean my hands (such as hand sanitizer)	☐	☐	☐	☐
I use disinfectant sprays or wipes to clean frequently touched surfaces and objects	☐	☐	☐	☐
I stay home when I feel unwell or I would stay home if I had felt unwell	☐	☐	☐	☐
I avoid shaking hands or hugging anyone who doesn't live with me	☐	☐	☐	☐
I keep a safe distance (at least 2 metres) from people outside of the home when possible	☐	☐	☐	☐
I avoid crowded places	☐	☐	☐	☐
I minimize the number of trips to supermarkets, pharmacies, and other essential businesses	☐	☐	☐	☐
I send someone else to shop for groceries and other essentials or use a delivery service	☐	☐	☐	☐
I disinfect groceries and other essential items when they come into my home	☐	☐	☐	☐
I avoid meeting with friends or family who do not live with me	☐	☐	☐	☐
I meet friends and family who do not live with me outdoors where we can physically distance	☐	☐	☐	☐

I meet friends and family who do not live with me indoors, when we are six or less people	☐	☐	☐	☐
I avoid restaurants and bars that lack outdoor spaces	☐	☐	☐	☐
I avoid restaurants	☐	☐	☐	☐
I avoid bars	☐	☐	☐	☐
I avoid in-person religious services (in congregation)	☐	☐	☐	☐
I avoid large public gatherings, such as weddings, protests, and festivals	☐	☐	☐	☐

37. How often have you worn a face mask that covers the nose and mouth since June 2020? June is when the use of face masks began to be recommended by public health organizations, such as the World Health Organization.

	**Never**	**Rarely**	**Often**	**Very frequently**	**I have not gone to this type of place**
**In an outdoor public place, such as a park or on a street, when physical distancing is not possible**	☐	☐	☐	☐	☐
**In an indoor public place, such as a grocery store, healthcare facility, or recreational facility**	☐	☐	☐	☐	☐
**On public transportation, including buses, taxis, trains, and others**	☐	☐	☐	☐	☐
**In a private outdoor place, such as a friend's yard, when physical distancing is not possible**	☐	☐	☐	☐	☐
**In a private indoor place, such as a friend's house**	☐	☐	☐	☐	☐

38. Have you travelled outside of British Columbia or been in close contact with someone who travelled outside of British Columbia since March 2020 (please check (✓) all that apply):

☐ Yes, I travelled outside of Canada

☐ Yes, I travelled outside of British Columbia, but not outside of Canada

☐ Yes, I was exposed to someone who travelled outside of British Columbia (either to another province/territory or country)

☐ No, I haven't travelled outside of the province

☐ I don't know

39. If you have been tested for COVID-19, please specify the reason why. Here, testing refers to the nasal swab or the saline gargle tests. (Please check (✓) all that apply):

☐ I have not been tested for COVID-19

☐ I may have been exposed to someone with COVID-19

☐ I am a health care professional

☐ I had recently travelled outside of Canada

☐ I called 8-1-1 and they advised me to get tested

☐ I used an online symptoms assessment tool and was advised to get tested

☐ I was symptomatic and went to a testing site

☐ I was tested while in the hospital for a different health concern

☐ I was being monitored through a symptom checking application and was sent for testing

☐ I was tested as part of a travelling requirement (for example, other countries may require proof of a negative COVID-19 test before flying into their airports)

☐ Other: ________________________

40. Since March 2020, have you gone to a hospital, medical clinic, or other medical or laboratory facility either for yourself, or to accompany / visit another person, <u>for health reasons</u> (not for employment), such as a medical reason or a test?

☐ Yes

☐ No

☐ I don't know

Section 6: Beliefs

In this section, we are interested in your beliefs about COVID-19. There are no right or wrong answers and your answers will remain confidential. Please choose the answer that most fits how you feel about the statement.

41. To what extent do you agree with the following statements about the COVID-19 pandemic?

	Strongly disagree	Slightly disagree	Neutral	Slightly agree	Strongly agree
**If I follow precautions, I will be less likely to get COVID-19.**	☐	☐	☐	☐	☐
**I am motivated to follow precautions specifically because my friends and/or family follow precautions.**	☐	☐	☐	☐	☐
**I am motivated to follow the precautions because it is the "right thing to do".**	☐	☐	☐	☐	☐
**I feel that I am more vulnerable to COVID-19 because of a medical condition.**	☐	☐	☐	☐	☐
**I should follow the precautions because I have a greater chance of getting COVID-19 for reasons other than a medical condition, such as race, age, or housing.**	☐	☐	☐	☐	☐
**COVID-19 is just as bad as the flu so I don't need extra precautions.**					
**I don't think I would experience serious outcomes, such as hospitalization, if I contracted COVID-19.**					

Section 7: Final Questions

Thank you for completing this survey. Your responses will help us understand the burden of COVID-19 among people living in British Columbia.

42. Would you be interested in receiving information about the results of this study?

☐ Yes
☐ No

43. Would you be interested in receiving information about participating in other health-related research conducted by researchers at Arthritis Research Canada?

☐ Yes
☐ No

44. If you answered YES to questions 42 or 43 above, please provide your e-mail address below in order for us to contact you about this study or future health-related research studies that you may be interested in:

E-mail address: ____

45. If you have any other questions or comments, please leave them below:

____
____
____
____
____
____
____

Thank you for taking the time to complete this survey.
Please return it in the enclosed postage-paid envelope.
