# Supplementary material for: Immunosuppression and COVID-19 infection in British Columbia: Protocol for a linkage study of population-based administrative and self-reported survey data
Source: PLoS One. 2021 Nov 19;16(11):e0259601. doi: 10.1371/journal.pone.0259601 (PMC8604283; doi:10.1371/journal.pone.0259601)
Supplement: S4 Appendix — (PDF) [file pone.0259601.s004.pdf]

General cognitive probes:

1. Why did you give that answer?
2. What did that question mean to you?
3. What do you understand by “X”? What might “not X” look like?
4. What does “X” mean to you in this context?
5. For questions with a time component: Is it easy or hard to remember, given that it was “Y” amount of time ago?
6. Do you find it easy to understand the scale/homunculus?
7. Did you find it easy to understand the question? I noticed that you hesitated at question “X”.
8. Are you happy with all the responses or some you thought maybe you could have ticked a different box?
9. Was it easy to think of your answer?
10. What do you think the top/bottom of the scale/homunculus means?
11. Which of the questions in this section were the most difficult to answer or the most confusing?
12. Were there any response definitions you felt were missing?
13. How do you <u>feel</u> about answering this question?
14. What does (vague medical term) mean to you?
15. Did you feel like any of the question stems were too long/confusing to read?
16. Are there any health-related behaviors or lifestyle factors that you feel should be included?
17. Did you feel comfortable answering

Item-specific questions:

1. How did you find the format of the question that asks about your household members
2. Did you feel comfortable answering the question about your ethnic background?
3. Did you feel comfortable answering the question about your income bracket?
4. How did you define religion and spirituality for yourself?
5. Was it easy to think about your vaccinations, if not how did you recall them?
6. Did you find it easy to understand the pandemic phases?
7. How did you define essential work?
8. How did you interpret vulnerability to COVID-19?
